# Evaluation of light microscopy and rapid diagnostic test for the detection of malaria under operational field conditions: a household survey in Ethiopia

**DOI:** 10.1186/1475-2875-7-118

**Published:** 2008-07-03

**Authors:** Tekola Endeshaw, Teshome Gebre, Jeremiah Ngondi, Patricia M Graves, Estifanos B Shargie, Yeshewamebrat Ejigsemahu, Berhan Ayele, Gedeon Yohannes, Tesfaye Teferi, Ayenew Messele, Mulat Zerihun, Asrat Genet, Aryc W Mosher, Paul M Emerson, Frank O Richards

**Affiliations:** 1 The Carter Center, Addis Ababa, Ethiopia; 2 The Carter Center, Atlanta, Georgia, USA; 3 Amhara Regional Health Bureau, Bahir Dar, Ethiopia; 4 Department of Public Health and Primary Care, University of Cambridge, Cambridge, UK

## Abstract

**Background:**

In most resource-poor settings, malaria is usually diagnosed based on clinical signs and symptoms and not by detection of parasites in the blood using microscopy or rapid diagnostic tests (RDT). In population-based malaria surveys, accurate diagnosis is important: microscopy provides the gold standard, whilst RDTs allow immediate findings and treatment. The concordance between RDTs and microscopy in low or unstable transmission areas has not been evaluated.

**Objectives:**

This study aimed to estimate the prevalence of malaria parasites in randomly selected malarious areas of Amhara, Oromia, and Southern Nations, Nationalities and Peoples' (SNNP) regions of Ethiopia, using microscopy and RDT, and to investigate the agreement between microscopy and RDT under field conditions.

**Methods:**

A population-based survey was conducted in 224 randomly selected clusters of 25 households each in Amhara, Oromia and SNNP regions, between December 2006 and February 2007. Fingerpick blood samples from all persons living in even-numbered households were tested using two methods: light microscopy of Giemsa-stained blood slides; and RDT (ParaScreen device for Pan/Pf).

**Results:**

A total of 13,960 people were eligible for malaria parasite testing of whom 11,504 (82%) were included in the analysis. Overall slide positivity rate was 4.1% (95% confidence interval [CI] 3.4–5.0%) while ParaScreen RDT was positive in 3.3% (95% CI 2.6–4.1%) of those tested. Considering microscopy as the gold standard, ParaScreen RDT exhibited high specificity (98.5%; 95% CI 98.3–98.7) and moderate sensitivity (47.5%; 95% CI 42.8–52.2) with a positive predictive value of 56.8% (95% CI 51.7–61.9) and negative predictive value of 97.6% (95% CI 97.6–98.1%) under field conditions.

**Conclusion:**

Blood slide microscopy remains the preferred option for population-based prevalence surveys of malaria parasitaemia. The level of agreement between microscopy and RDT warrants further investigation in different transmission settings and in the clinical situation.

## Background

Malaria is one of the leading public health problems in Ethiopia. About 75% of the total area of the country is malarious, with more than two thirds of the total population estimated to be at risk of infection [1,2]. Malaria transmission in Ethiopia is seasonal, depending mostly on altitude and rainfall. The two main seasons for transmission of malaria in Ethiopia are September to November, sometimes extended to December after heavy summer rains, and March to May, after the light rains [3-5]. Malaria epidemics are relatively frequent [6,7] involving highland or highland fringe areas, mainly areas 1,000–2,000 meters above sea level, in which the population lacks immunity to malaria [3,8,9]. In Ethiopia, *Plasmodium falciparum *and *Plasmodium vivax *account for about 60% and 40% of infections, respectively, during the peak transmission period [3,10].

Early diagnosis and prompt treatment is one of the key strategies for malaria control. Clinical diagnosis is widely used in areas where laboratory facilities are not available; however, it is unreliable due to the non-specific nature of signs and symptoms of malaria [10,11]. Microscopy still remains the gold standard for laboratory diagnosis of malaria, although it is not accessible and affordable in most peripheral health facilities. Recent advent of rapid diagnostic tests (RDT) for malaria may be a significant step forward in case detection, management and reduction of unnecessary treatment. Such RDT could also be useful in malaria diagnosis during population-based surveys and to provide immediate treatment based on the results. However, the accuracy of RDT under field conditions in low transmission areas remains questionable [11].

There are numerous malaria rapid diagnostic tests that are commercially available [12], all of which detect malaria antigen in blood flowing along a membrane containing specific anti-malaria antibodies. The tests fall into a few basic types depending on which antigen is targeted. Most tests which detect *P. falciparum *are based on the histidine-rich protein 2 (HRP-2), which is specific to that species. Other tests detect the parasite enzyme lactate dehydrogenase (LDH), using either monoclonal antibodies which react with LDH of all species including *P. falciparum *(so-called PAN or pLDH), or antibodies specific for *P. falciparum *LDH. Other antigens including aldolase (which can distinguish non-*P. falciparum *from mixed infections) and other *P. vivax *specific tests are in early development or use. A distinction between the HRP-2 and LDH based tests is that HRP-2 may persist in the blood stream for days or weeks after treatment, whereas LDH is only detected if live parasites are present.

In addition to variation in antigen detected, the tests are available in many formats including plastic cassettes, cards or dipsticks, and quality depends on manufacturer as well as storage conditions. Recent reviews from clinical trials have found that HRP-2 based *P. falciparum*-specific tests generally have greater sensitivity (over 90%) than the pLDH-based tests when compared with microscopy in clinical cases, whilst sensitivity of pLDH tests for non-*P. falciparum *species was low [13-15]. Specificity of both types of tests was reported to be good (>85%). Despite the encouraging results from the RDT trials in clinical cases, there is limited information about their accuracy and predictive value in population based surveys of malaria prevalence, where people may not have clinical signs and parasitaemia is likely to be lower than in a clinic setting. In field conditions, RDTs may be exposed to leads of heat and humidity greater than those recommended by the manufacturer.

The Carter Center is one of the partners in malaria control in Ethiopia and is committed to integrated malaria control with trachoma (Amhara region) and onchocerciasis (Oromia and SNNP regions). The Carter Center contributed three million long-lasting insecticidal nets (LLIN) in 2007 to the total 20 million distributed nationally in the last three years [16]. The purpose of this study was to collect baseline information on the prevalence of malaria parasites at a community level in three regions of Ethiopia, prior to mass distribution of LLINs. The gold standard for determining parasite prevalence was thick and thin blood films, stained with Giemsa and examined at 1,000× magnification. In the field ParaScreen RDT, which detects both *P. falciparum *using HRP-2 and all species (PAN) using pLDH, was used so that immediate treatment could be offered for those with positive tests. This design enabled evaluation of the agreement between ParaScreen RDT and microscopy diagnoses of malaria under field conditions.

## Methods

### Study settings and study population

This is a population based cross-sectional survey that was conducted in three regions of Ethiopia (Amhara, Oromia and SNNP) between mid-December, 2006 and mid-February, 2007. The sample size estimation and sample selection process have been described elsewhere [16]. Briefly, a multistage cluster random sampling design was used to select 224 clusters, and 25 households were randomly selected in each cluster. Clusters were defined as *kebeles *(the smallest administrative unit with an average population of 5,000). All consenting residents (all age groups and both sexes) of even- numbered households had their blood tested for malaria parasites.

### Malaria parasite detection

In the field, blood samples collected from the study participants were tested for malaria parasites using ParaScreen RDT (Zephyr Biomedical Systems, Verna, Goa, India). ParaScreen is an immunochromatographic test that detects the presence of pan malaria specific antigen (pLDH) for the detection of all non-falciparum malarial parasites whereas the detection of *P. falciparum *utilises recognition of specific histidine rich protein-2 (HRP-2). The test uses approximately 5 μl of blood and is readable after 15 minutes following the manufacturer's instructions. Participants with positive RDT results were offered immediate treatment according to national guidelines: CoArtem^® ^for *P. falciparum *infection; chloroquine for other *Plasmodium *infection; and clinic-based quinine therapy for pregnant women [10].

In addition, two blood slides, each composed of thick and thin films, were prepared for each participant by a medical laboratory technician according to standard WHO-approved protocol [17]. Slides were labelled and air-dried horizontally in a slide tray in the field. Thin films were fixed immediately after drying with methanol. Slides were stained with 3% Giemsa at the nearest health facility at the end of the day. Usually, field teams returned to the clinic each evening but when working in inaccessible areas, which required walking up to eight hours each way, they were obliged to sleep in the field and stain the slides the following day. To ensure maximum participation, households with absentees were revisited up to two times on the same day.

Blood slides were read at a reference laboratory in Addis Ababa and classified qualitatively as either negative, *P. falciparum*-positive, *P. vivax*-positive, or mixed infection. One hundred high power fields of the thick film were examined at a magnification of 1,000×, before identifying a slide as negative or positive. If positive, the thin film was read to determine the species. Parasite density was not quantified. To ensure accuracy, all positive slides and a random sample (5%) of the negative slides were re-examined by a separate expert microscopist, who was blinded to the diagnosis of the first slide-reader. The level of agreement between first and second readings was 99.4%. The second slide from each participant was read if the first was broken or unreadable. The identity of survey participants who had positive blood slides was sent back to the field teams for follow-up and appropriate treatment, where necessary.

### Statistical analysis

Statistical analysis was conducted using Stata 8.2 (Stata Corporation, College Station, Texas). Distribution of participants' characteristics and malaria parasite prevalence were assessed using contingency tables while differences in proportions were compared using the chi square test. Taking blood slide microscopy as the gold standard, the performance of the rapid diagnostic test was compared to generate summary statistics for diagnostic tests using the *diagt *module in Stata [18].

### Ethical consideration

The protocol received ethical approval from the Emory University Institutional Review Board (IRB 1816) and the Amhara, Oromia and SNNP Regional Health Bureaus. Signed informed consent was sought from each individual and parents of children aged under 18 years and signed assent was sought from children older than 10 years in accordance with the tenets of the declaration of Helsinki for blood films. Personal identifiers were removed from the data set before analyses were undertaken.

## Results

**Figure 1:**
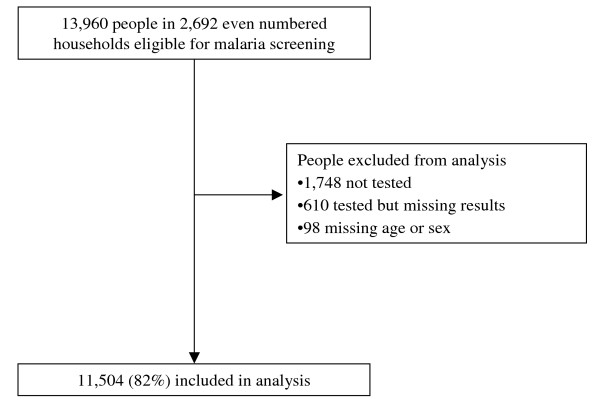
The sample population.

The demographic characteristics of the study participants are shown in Table 1. A total of 13,960 people in 2,692 households were enumerated of whom 2,456 (18%) were excluded from analysis due to missing data (Figure 1). Of the 11,504 people included in the analysis, 53.8% were female and the overall mean age was 20.7 years.

**Table 1:** Demographic characteristics of the study participants in three regions

Region	Male n (%)	Female n (%)	Total N	Age mean (SD)
Amhara	3,495 (45.6)	4,169 (54.4)	7,664	20.7 (17.7)
Oromia	921 (46.5)	1,061 (53.5)	1,982	18.1 (16.9)
SNNP	897 (48.3)	961 (51.7)	1,858	19.5 (16.1)
				
Total	5,313 (46.2)	6,191 (53.8)	11,504	20.7 (17.7)

SD, standard deviation

The malaria parasite prevalence by blood slide microscopy and ParaScreen RDT are shown in Table 2. The prevalence by blood slide microscopy overall was 4.1% (95% CI 3.4 – 5.0%) and by Para Screen RDT it was 3.3% (95% CI 2.6–4.1%). By region, the prevalence by blood slide was 4.6% (95% CI 3.8–5.6) in Amhara, 0.9% (95% CI 0.5–1.6) in Oromia, and 6.1% (95% CI 4.5–8.5) in SNNP. The prevalence of malaria parasites by blood slide microscopy was higher than prevalence by ParaScreen RDT in Amhara (P = 0.001) and lower than RDT in Oromia (P < 0.001). There was no statistically significant difference between the two diagnostic methods in SNNP (P = 0.3).

**Table 2:** Malaria prevalence by region based on blood slide microscopy and Rapid Diagnostic Test (ParaScreen)

Region	Number tested	Blood slide microscopy				Rapid diagnostic test (ParaScreen)		
			
		Pf %	Pv %	Pf & Pv %	Total % (95%CI)	Pan/Pf %	Pan %	Total % (95%CI)
Amhara	7,664	2.4	1.9	0.4	4.6 (3.8–5.7)	2.0	1.1	3.1 (2.4–3.9)
Oromia	1,982	0.7	0.1	0	0.9 (0.5–1.6)	1.5	1.2	2.6 (1.1–6.5)
SNNP	1,858	3.6	1.8	0	5.4 (3.4–8.5)	4.5	1.7	6.2 (3.6–10.2)
								
Total	11,504	2.2	1.6	0.3	4.1 (3.4–5.0)	2.1	1.1	3.3 (2.6–4.1)

Pf, *Plasmodium falciparum*; Pv, *Plasmodium vivax*

The sensitivity, specificity, positive predictive value and negative predictive value of ParaScreen RDT against blood slide microscopy for all species of malaria combined are shown in Table 3. The overall sensitivity was 47.5% (95% CI 42.8–52.2) and ranged from 41.0% in SNNP to 50.0% in Oromia. Specificity of ParaScreen RDT was 98.5% overall and ranged from 95.7% in SNNP to 99.4% in Amhara. The overall positive predictive value was 56.8% (95%CI 51.7–61.9) and ranged from 36.1% to 77.8% depending on region, while negative predictive value was very high (97.9%).

**Table 3:** Sensitivity, specificity, positive predictive value and negative predictive value of blood slide microscopy compared to Rapid Diagnostic Test (ParaScreen)

Region	Sensitivity		Specificity		Positive predictive value		Negative predictive value	
				
	%	95%CI	%	95%CI	%	95%CI	%	95%CI
Amhara	49.4	(43.9–54.9)	99.4	(99.1–99.5)	77.8	(71.6–83.0)	97.7	(97.4–98.1)
Oromia	50.0	(24.7–75.3)	97.9	(97.2–98.5)	16.3	(7.3–29.7)	99.6	(99.2–99.8)
SNNP	41.0	(31.5–51.0)	95.7	(94.6–96.6)	36.1	(27.5–45.4)	96.4	(95.5–97.3)
								
Total	47.5	(42.8–52.2)	98.5	(98.3–98.7)	56.8	(51.7–61.9)	97.9	(97.6–98.1)

The sensitivity and specificity of ParaScreen RDT compared to blood slide microscopy for the different malaria species are shown in Table 4. Because both the Pf-specific and the 'PAN' antigen used by the ParaScreen RDT are detected in *P. falciparum *infections, giving the result "Pf plus PAN" even for non-mixed *P. falciparum *infections, it was not possible to directly compare the performance of the test against *P. falciparum *single infections detected by slide. Therefore, the sensitivity and specificity of "Pf or mixed" (by slide) to "Pf plus PAN" (by RDT) were compared; whilst non-*P. falciparum *infections detected by slide were compared directly to the "PAN only" RDT results.

**Table 4:** Comparison of prevalence, sensitivity and specificity of blood slide microscopy and Rapid Diagnostic Test (ParaScreen) by species detected (N = 11,504)

Species	Prevalence		Sensitivity		Specificity	
			
	Positive by BS %	Positive by RDT %	%	95%CI	%	95%CI
Pf or mixed (by BS) Vs. Pf/Pan (by RDT)	2.5	2.1	51.4	(45.4–57.5)	99.0	(98.8–99.2)
Non-Pf (by BS) Vs. Pan only (by RDT)	1.6	1.1	30.7	(24.1–38.0)	99.4	(99.2–99.5)
All species	4.1	3.3	47.5	(42.8–52.2)	98.5	(98.3–98.7)

BS, blood slide; Pf, *Plasmodium falciparum*; RDT, rapid diagnostic test

The sensitivity of the ParaScreen RDT for *P. falciparum *or mixed infection compared to slide microscopy was 51.4% (95% CI 45.5–57.5) (Table 4). Much lower sensitivity of 30.7% (95% CI 24.1–38.0) was found for non-*P. falciparum *species. However, specificity was very high for both species: 99.0% (95% CI 98.8–99.) for *P. falciparum *or mixed and 99.4% (95% CI 99.2–99.5) for non-*P. falciparum *malaria.

## Discussion

This large population based survey was conducted during the late transmission season during a non-epidemic year for malaria in Ethiopia. The study used the conventionally accepted standard malaria diagnostic method, light microscopy of peripheral blood slides, and a new technique (ParaScreen rapid diagnostic test, RDT). Overall, more infections were detected by blood slide microscopy (4.1%) than by ParaScreen RDT (3.3%), although the difference was not statistically significant. However, by region, significantly more infections were detected by RDT in Oromia and significantly fewer by RDT in Amhara.

Despite little difference between the overall prevalence of malaria detected by the two tests, the overall sensitivity of ParaScreen that we observed was low (47.5%). This indicates that there was substantial non-overlap between the positives detected by each test. The positive predictive value was correspondingly low (56.8%). Surprisingly, SNNP region had a positive predictive value of only 36.1% (95% CI 27.5–45.4) which was lower than expected given that SNNP had the highest prevalence of malaria parasites based on microscopy as the gold standard.

Reports from elsewhere indicated that RDTs have shown a comparable level of accuracy to microscopy in clinical settings [19,20]. The sensitivity of the RDTs observed in our study is much lower than predicted by previous studies [13-15]. This difference may arise for two reasons: firstly, it is possible that parasitaemias were very low in people not seeking treatment; and secondly, the RDTs were possibly defective or handled inappropriately causing them to lose sensitivity. Light microscopy can routinely detect parasitaemia levels as low as 40 parasites/μl, and experienced microscopists can detect as low as 5–10 parasites/μl of blood [11], whereas RDTs usually have a capacity to detect 100 parasites/μl of blood [11,19]. The lack of sensitivity of RDTs at low parasitaemia compared to microscopy is one of the shortcomings noted elsewhere [11,19,20], which is possibly also reflected in our findings. Regarding the quality of RDTs, the diagnostic accuracy can be affected by several factors such as quality of the products, storage temperature and humidity, and end users' performance [11,21]. The RDTs used in this study were manufactured in May and June 2006 with an expiry date of April 2008 suggesting that they should have been in a good condition during the survey field work (mid-December 2006 to mid February, 2007). However, the results suggest that an appropriate quality control scheme should accompany any effort to initiate the use of RDT at a population based scale, especially in remote settings.

In general, the prevalence of malaria showed significant variation among the regions surveyed, but the overall prevalence was low. As would be expected, two malaria parasite species, *P. falciparum *and *P. vivax *were identified by microscopy in this study. In Ethiopia, the two predominant malaria species recorded are *P. falciparum *(~60%) and *P. vivax *(~40%) [3,22,23]. However, this proportion can change during hot-dry seasons following the peak transmission period, when more relapse cases could be expected due to *P. vivax *infection, [24]. The preponderance of one malaria species over the other at a particular period might vary from one area to another, not only depending on climatic and seasonal factors but also owing to variation in geographical localities [25].

Even though clinical history of the participants was not recorded in our study, evidence from other studies showed that RDT positive cases missed by microscopy might be individuals who had been treated but in whom antigenemia persists [20,21]. ParaScreen RDT exhibited more sensitivity to *P. falciparum *or mixed infections than to non-*P. falciparum *(most likely *P. vivax*) infection. In addition to intrinsic lower sensitivity of the antigen used, this might be attributed to the longer persistence of *P. falciparum *antigen (HRP-2) after treatment or resolution than pLDH antigen [21,26]. Other studies have also demonstrated that the HRP-2 assay showed more sensitivity compared to the pLDH antigen based assay due to quick clearance of the latter antigen after treatment [27].

In this study, quantitative parasite counts were not conducted; nonetheless, there were cases with high loads of malaria parasites with asexual stages (ranging ++ to ++++ per high power field) which turned out to be negative by RDT. This phenomenon suggests the possibility of under diagnosis of malaria parasites by RDT [19] and requires further investigations in the Ethiopian context. A similar observation was reported from other settings where RDT showed false negative results in the presence of high parasitaemia [25] implying the need for a continuous quality assurance system to be instituted at every step before disseminating the RDT products to the end users.

## Conclusion

Based on the results of this study, well-conducted blood slide microscopy for malaria diagnosis for population-based surveys remains the preferred option. The level of the agreement between RDT and light microscopy for malaria diagnosis warrants further investigations in clinical facilities in the Ethiopian context.

## Abbreviations

CI: confidence interval; HRP-2: histidine-rich protein 2; LDH: lactate dehydrogenase; LLIN: long-lasting insecticidal nets; RDT: rapid diagnostic test; SNNP: Southern Nations Nationalities and Peoples'; WHO: World Health Organization.

## Competing interests

The authors declare that they have no competing interests.

## Authors' contributions

PME, EBS, PMG, YE, TG, AWM, and FOR designed the survey; TE, EBS, YE, TG, GY, BA, TT, MZ, AM, AG and AWM supervised and conducted field work; TE, BA, GY and EBS supervised and conducted microscopy; JN, PMG and YE supervised data management, cleaning, and production of the analysis data set; JN, TE and PMG conducted the analysis; TE, JN, and PMG drafted the manuscript which all authors edited and approved.
